# Beyond the Firewall: A Multistakeholder Simulation for Cybersecurity Response in Health Information and Informatics Practice

**DOI:** 10.63116/AH.000000004

**Published:** 2026-08-12

**Authors:** Shannon H. Houser, Cathy A. Flite, Susan L. Foster

**Affiliations:** 1 Health Services Administration Department, University of Alabama at Birmingham; 2 Health Services Administration and Policy Department, Temple University; 3 Health Information Management Department, Florida SouthWestern State College

**Keywords:** breach response, cybersecurity, data governance, experiential learning, ransomware, simulation-based learning

## Abstract

**Background:**

Cybersecurity incidents, particularly ransomware attacks, pose significant threats to health care systems by disrupting operations, compromising protected health information, and undermining patient trust. Health information management and informatics professionals play a central role in responding to these incidents; however, educational preparation often emphasizes theoretical knowledge with limited opportunities for applied, practice-based learning. This study aims to describe the design, implementation, and evaluation of a multistakeholder tabletop simulation to enhance competencies in cybersecurity incident response, regulatory compliance, and interdisciplinary collaboration.

**Methods:**

A simulation-based educational intervention was implemented in 2 settings, namely, a national professional conference workshop and an undergraduate health information management classroom at a large public research university. The simulation was designed as a multistakeholder tabletop exercise set in a 350-bed acute care hospital experiencing a ransomware attack affecting key systems, including the electronic health record and patient portal. Participants assumed interdisciplinary roles representing leadership, clinical, technical, compliance, and vendor perspectives. A postsimulation survey assessed perceptions of realism, clarity of objectives, debriefing effectiveness, and learning outcomes. Descriptive statistics were used to summarize the responses.

**Results:**

Participants in both groups strongly agreed that the simulation was realistic, well structured, and effective at reinforcing key learning objectives. The simulation enhanced participants’ understanding of cybersecurity response processes, privacy and security principles, and interdisciplinary roles and responsibilities. Although responses were consistently positive, variation between professional and student participants reflected differences in experience and familiarity with health care operations.

**Conclusions:**

This study demonstrates that tabletop simulation is an effective, practice-oriented approach to preparing health information management and informatics learners to respond to complex cybersecurity incidents. By integrating technical, regulatory, and leadership perspectives within a realistic health care context, the simulation supports applied learning and workforce readiness. Simulation-based approaches offer a scalable strategy for strengthening cybersecurity preparedness in health information management and informatics education.

## Introduction and Background

Cybersecurity incidents, particularly ransomware attacks, have become one of the most significant threats to health care systems, disrupting clinical operations, compromising protected health information (PHI), and undermining patient trust. A 2024 Sophos survey reported that 67% of health care organizations experienced a ransomware attack, with 53% of affected organizations paying a ransom to recover data, highlighting the sector’s vulnerability.^1^ Health care organizations are especially attractive targets because of the high value of patient demographic, clinical, and financial data stored across electronic health records (EHRs), cloud environments, and interconnected digital systems.^2^ Cybercriminals exploit these environments using techniques such as malware, phishing, and structured query language (SQL) injection, as well as zero-day exploits, resulting in significant operational disruptions and long-term reputational damage.^3^^,^^4^

Recent large-scale incidents further illustrate the systemic impact of cyberattacks. The 2024 ransomware attack on UnitedHealth Group subsidiary Change Healthcare disrupted claims processing nationwide, significantly affecting reimbursement workflows and placing financial strain on health care providers, particularly those in rural and underserved areas.^5^ Beyond these operational consequences, cybersecurity incidents directly affect patient care and safety. Evidence suggests that cyberattacks can disrupt health care delivery through electronic system downtime, cancellation of scheduled care, or ambulance diversion, underscoring the clinical implications of digital system disruptions.^6^ In addition, data breaches erode patient trust and may reduce patients’ willingness to engage with digital health platforms, further affecting care delivery and information exchange.^7^

These challenges position health information and informatics professionals (HIIPs) at the forefront of cybersecurity response and data governance in modern health care organizations. Their responsibilities extend beyond technical mitigation to encompass the following: ensuring data integrity and availability, managing access to health information, supporting breach investigation and documentation, and coordinating communication with internal and external stakeholders. Compliance with regulatory frameworks, including the Health Insurance Portability and Accountability Act (HIPAA) Breach Notification Rule (45 CFR §§ 164.400–414)^8^^,^^9^ and the Health Information Technology for Economic and Clinical Health (HITECH) Act, requires timely risk assessment, reporting, and mitigation of PHI exposure.^10^ Additional compliance obligations may arise because of state laws. In practice, HIIP must also align organizational responses with established frameworks, such as the National Institute of Standards and Technology (NIST) Cybersecurity Framework, which emphasizes identifying, protecting, detecting, responding to, and recovering from cybersecurity incidents.^11^ Additional guidance from the Health Sector Cybersecurity Coordination Center^12^ and the Cybersecurity and Infrastructure Security Agency^13^ further supports organizations in addressing evolving cybersecurity threats.

Despite the critical role of HIIP in managing cybersecurity incidents, education and training continues to evolve to better prepare students for professional practice. Many academic programs emphasize foundational knowledge of privacy, security, and compliance but provide limited opportunities for learners to apply these concepts in realistic, high-pressure, and interdisciplinary scenarios. Systematic reviews of cybersecurity training approaches further highlight the need for more applied, scenario-based learning methods to effectively prepare learners for real-world cyber threats.^14^ As a result, gaps remain in learners’ ability to integrate cybersecurity response, regulatory compliance, and cross-functional coordination in real-world practice settings.^15^ This disconnect highlights the need for educational approaches that move beyond theoretical instruction and support applied, practice-based competency development.

In addition, recent work has further emphasized the evolving role of HIIP in addressing emerging technologies and associated risks, including cybersecurity and data governance challenges.^16^ As digital health technologies such as telehealth platforms expand, new privacy and security vulnerabilities continue to emerge, highlighting the need for workforce preparation that integrates technical, regulatory, and operational competencies.^17^ These trends reinforce the importance of educational approaches that prepare learners to navigate complex, technology-driven health care environments.

Simulation-based experiential learning offers a practical and scalable approach to addressing this gap. Grounded in Kolb’s experiential learning model—comprising concrete experience, reflective observation, abstract conceptualization, and active experimentation—simulation immerses learners in realistic scenarios that promote active decision-making and skill development.^18^ Prior studies have also demonstrated that interactive, simulation-based approaches, including tabletop exercises and escape room activities, enhance student engagement and applied learning in health administration education.^19^ Emerging evidence further demonstrates that simulation-based training improves confidence, collaboration, and retention of cybersecurity practices among health care professionals.^20–22^ Recent work has also highlighted the effectiveness of tabletop cybersecurity simulations in preparing health informatics learners for real-world incident response.^23^ Within health information and informatics education, simulation provides a mechanism to operationalize complex concepts, such as data governance, breach response, and regulatory compliance, in a controlled yet realistic environment.

Taken together, these trends underscore the need for interdisciplinary, practice-oriented training approaches that prepare HIIP to manage complex cybersecurity incidents. Integrating simulation into health information and informatics education can bridge the gap between theoretical knowledge and real-world application, supporting workforce readiness in an increasingly digital and risk-intensive health care environment.

To address this need, this study describes the design, implementation, and evaluation of a multistakeholder cybersecurity simulation developed for health information and informatics learners. The simulation was designed to support practice-based competency development by integrating cybersecurity incident response, regulatory compliance, and interdisciplinary leadership in a realistic health care setting.

## Methods

### Study Design

This study employed a simulation-based educational intervention to evaluate the effectiveness of a tabletop cybersecurity exercise designed for health information and informatics learners. The study utilized a descriptive design to assess participant engagement, perceived learning, and applicability of the simulation to real-world practice settings.

The simulation was developed to address the growing need for practical cybersecurity training in health care by immersing participants in the complexities of managing a ransomware attack in a health care environment. The simulation provided a structured, interdisciplinary learning experience in which participants applied critical thinking, regulatory knowledge, and leadership skills to navigate a rapidly evolving digital crisis.

The design of the simulation was grounded in Kolb’s experiential learning cycle, emphasizing iterative learning through concrete experience, reflective observation, abstract conceptualization, and active experimentation.^18^ Consistent with this framework, the simulation focused on 3 core competency domains relevant to health information and informatics management practice, namely, cybersecurity incident management, regulatory compliance, and leadership during crisis situations.

This study evaluated the implementation of the simulation in 2 distinct settings—a professional conference and an academic classroom—to assess its applicability across diverse learner populations.

### Simulation Design

The tabletop simulation was developed to enhance cybersecurity preparedness and interdisciplinary crisis management competencies among health information management and informatics learners. The simulation was designed as a structured, immersive learning experience in which participants engaged in realistic, high-pressure scenarios involving health care data breaches. It is adaptable for implementation in both academic and professional settings and can be conducted within a single session or divided into multiple instructional modules, allowing flexibility based on course objectives, learner level, and available instructional time. Unlike prior tabletop cybersecurity simulations that primarily target health informatics learners,^23^ this simulation incorporates a multistakeholder framework representing administrative, clinical, technical, legal, and vendor stakeholders, reflecting the complexity of real-world health information management and informatics practice.

The simulation was centered on a realistic health care scenario involving a mid-sized, 350-bed acute care hospital that was experiencing a ransomware attack. The incident disrupted access to multiple digital systems, including the EHRs, telehealth platforms, and patient portals, resulting in compromised clinical workflows, challenges in maintaining patient care delivery, and increased organizational risk related to regulatory compliance, legal exposure, operational recovery, and public trust. This scenario was intentionally designed to reflect the complexity and urgency of real-world cybersecurity incidents in health care environments.

To enhance realism and applicability to practice, the simulation incorporated multiple incident pathways, including system-wide ransomware disruption and patient portal compromise resulting from phishing attacks. These scenarios required participants to assess risk, prioritize response actions, and coordinate interdisciplinary decision-making under time constraints. The simulation emphasized the integration of cybersecurity response processes with regulatory requirements, including HIPAA and HITECH breach notification considerations.

To support replication and instructional use, detailed scenario descriptions, response steps, and guided discussion questions used in the simulation are provided in Appendix A.

### Participants and Setting

The simulation was implemented across 2 distinct participant groups to evaluate its applicability in both professional and academic settings. The first group consisted of participants attending the national Assembly on Education (AOE) conference in October 2025, where the simulation was delivered as an in-person workshop. This group primarily included educators and professionals in health information and informatics. The second group consisted of senior undergraduate students enrolled in a health information management (HIM) operations management course at a large public research university during the Spring 2026 semester, where the simulation was conducted as part of an in-person classroom session.

Across both settings, participants engaged in the simulation through structured, role-based assignments designed to reflect real-world health care cybersecurity response teams. Participants were assigned interdisciplinary roles representing key stakeholders involved in managing a data breach, including executive leadership (eg, chief executive officer and chief operating officer), clinical leadership (eg, chief medical officer and nursing administrator), technical leadership (eg, chief information officer and chief information security officer), compliance and privacy officers, legal counsel, and external vendor representatives.

These roles were designed to reflect the responsibilities, decision-making processes, and communication challenges encountered in actual health care cybersecurity incidents. The role-based structure facilitated interdisciplinary collaboration and required participants to integrate administrative, clinical, technical, and regulatory perspectives in developing coordinated response strategies.

Table 1 presents the potential participant roles, primary responsibilities, and associated learning focus areas within the simulation. These roles emphasize leadership, regulatory compliance, clinical coordination, and information security practices, supporting the development of competencies relevant to health information and informatics practice.

**Table 1.** Potential Participant Roles, Responsibilities, and Learning Focus in the Simulation

**Role**	**Primary Responsibilities**	**Key Considerations/Learning Focus**
Chief executive officer (CEO)	Lead organization-wide crisis response; engage with board; approve communication strategies	Executive decision-making, strategic alignment, reputational risk management
Chief operating officer (COO)	Coordinate internal operations; maintain clinical and business continuity	Workflow recovery, communication with department heads, resource prioritization
Chief medical officer (CMO)	Monitor clinical impact of the breach; ensure patient care continuity and safety	Clinical operations, patient safety, cross-functional communication with administrative leaders
Nursing administrator	Lead nursing staff response; ensure continuity of bedside care and documentation workflows	Patient-centered operations, workforce mobilization, contingency planning in care delivery
Hospital administrator	Serve as internal liaison among departments; assist with logistics, escalation, and situational updates	Departmental coordination, administrative response planning, communication flow within hospital units
Chief information officer (CIO)	Oversee technical recovery; coordinate with IT and vendors	Data recovery, tech infrastructure, organizational communication
Health information management (HIM) Director	Assess patient record access and integrity; support breach notification	HIM functions in crisis, regulatory documentation, access and release under breach conditions
Chief compliance officer (CCO)	Ensure HIPAA/HITECH compliance; coordinate investigation and documentation	Regulatory strategy, interdepartmental collaboration, reporting workflows
Chief privacy officer (CPO)	Evaluate scope of PHI exposure and patient risk	Privacy risk mitigation, breach classification, policy adherence
Chief information security officer (CISO)	Lead cybersecurity incident investigation and containment; liaise with external cybersecurity partners	Threat detection, breach containment, cybersecurity strategy, forensic response planning
Public relations lead	Manage internal/external messaging; prepare media communications	Messaging consistency, public trust, stakeholder alignment
Health care attorney	Advise on legal exposure, liability, and reporting requirements	Legal risk analysis, documentation standards, breach response under legal constraints
Third-party vendor representative	Coordinate external IT service or telehealth vendor response	Vendor accountability, contract expectations, external partnership management

Abbreviations: PHI, protected health information; IT, information technology; HIPAA, Health Insurance Portability and Accountability Act; HITECH, Health Information Technology for Economic and Clinical Health Act.

### Simulation Procedures

The simulation was implemented using a structured 3-phase approach consisting of preparation, response planning, and debriefing. This phased design was intended to replicate the progression of real-world cybersecurity incidents while supporting experiential learning and interdisciplinary collaboration.

In the preparation phase, the instructor introduced the simulation scenario, assigned participants to interdisciplinary roles, and distributed a structured briefing packet. The briefing materials included an overview of the incident scenario, role descriptions, and relevant contextual information such as regulatory considerations and organizational challenges associated with the cybersecurity incident. This phase established the foundation for participant engagement and ensured a shared understanding of the scenario and participant expectations.

In the response planning phase, participants worked in role-based teams to assess the evolving cybersecurity incident and develop a coordinated response strategy under time constraints. Teams were expected to identify key priorities, evaluate risks, and formulate response plans addressing regulatory reporting requirements, communication strategies, system recovery, and leadership coordination. This phase emphasized real-time decision-making, interdisciplinary communication, and problem-solving in a simulated, high-pressure environment.

In the debriefing phase, participants presented their response strategies and engaged in a structured reflection facilitated by the instructor. The debrief focused on evaluating decision-making processes, identifying strengths and challenges in team collaboration, and connecting the simulation experience to real-world health information and informatics practice. This phase reinforced key learning objectives and promoted reflection on leadership, regulatory compliance, and organizational responses to cybersecurity incidents, with additional implementation and facilitation details provided in Appendix B.

### Evaluation and Measures

To evaluate the effectiveness and applicability of the simulation, a postsimulation survey was administered to participants across both implementation settings described in the Participants and Setting section. Data were collected using a standardized postsimulation feedback survey administered through Qualtrics. The survey captured participant perceptions of the simulation’s effectiveness, including engagement, perceived learning, and applicability to HIM and informatics practice. Demographic characteristics, such as age, gender, educational attainment, and race/ethnicity, were not collected because the survey was designed primarily to evaluate participant perception of the educational activity rather than to examine differences across demographic groups.

The survey instrument was designed to assess participant perception across several domains relevant to HIM and informatics practice, including perceived realism of the simulation scenario, clarity of simulation objectives, effectiveness of the debriefing process, and overall applicability to professional practice. Participants were asked to rate the extent to which the simulation reflected a realistic and plausible health care cybersecurity incident and whether the objectives were clearly defined. Additional items assessed perceived learning outcomes, including understanding of privacy and security principles and interdisciplinary roles and responsibilities during a cybersecurity incident. The survey also captured participant background characteristics, including professional experience and organizational affiliation for the professional/educator group.

Responses to structured items were measured using a 5-point Likert-type scale (1 = strongly agree to 5 = strongly disagree), enabling assessment of agreement levels across key domains. Lower mean scores indicate stronger agreement with survey items. Open-ended responses were also collected to provide qualitative insights into participant experiences.

### Data Analysis

Data analysis was conducted using descriptive statistical methods. Frequencies and percentages were calculated to summarize participant characteristics, response rates, and levels of agreement across the survey items. Mean scores and standard deviations were computed for Likert-scale items to assess overall trends and variability in participant perception.

Comparisons were made descriptively between the 2 participant groups (professional/educator group and student group) to examine differences in perceived realism, clarity of simulation objectives, and learning outcomes. No inferential statistical analyses were performed, as the study was designed for descriptive evaluation of the simulation.

### Ethical Considerations

This study reflects the evaluation of educational activities conducted in both a professional conference and classroom setting. The simulation and survey were implemented as part of instructional and professional development activities to assess participant feedback and learning experiences; therefore, this work is considered a program evaluation rather than human subjects research, and formal institutional review board review was not pursued.

## Results

### Participant Characteristics and Response Rates

A postsimulation survey was administered to 2 distinct groups, namely, participants attending the national AOE conference in 2025 and senior undergraduate students enrolled in a HIM course in Spring 2026.

At the AOE session, 87 participants attended and 53 completed the postsimulation survey, yielding a response rate of 61%. Among the respondents, 79% (n = 42) were affiliated with academic institutions (faculty, staff, or students), 11% (n = 6) represented health care organizations, and 10% (n = 5) were from the private sector, including consulting firms, technology companies, vendor organizations, and accrediting organization. All 18 undergraduate HIM students completed the survey, yielding a 100% response rate for the student group.

### Professional Experience in Health Information and Informatics

Among the AOE respondents, 75% (n = 40) reported more than 10 years of experience in health information or health informatics. Participants with 4-6 years of experience represented 9% (n = 5), followed by 7-10 years at 8% (n = 4) and 1-3 years at 6% (n = 3). One respondent reported no prior experience in health information or informatics. All student participants were senior-level undergraduate HIM students enrolled in a face-to-face operations management course.

### Perceived Realism of the Simulation

All AOE respondents (n = 53) indicated that the simulation scenario was realistic and plausible, with 62% (n = 33) strongly agreeing and 38% (n = 20) agreeing. The mean rating for realism among the professional/educator group was 1.38 (SD = 0.48). Among the student participants, 22% (n = 4) strongly agreed, 61% (n = 11) agreed, and 17% (n = 3) reported neutral responses. The mean rating for realism in the student group was 1.94 (SD = 0.62).

### Clarity of Simulation Objectives

Nearly all AOE respondents agreed that the simulation objectives were clearly defined, with 49% (n = 26) strongly agreeing and 49% (n = 26) agreeing. One respondent (2%; n = 1) reported a neutral response. The mean score for this item was 1.53 (SD = 0.54). Similarly, among the student participants, 33% (n = 6) strongly agreed, 61% (n = 11) agreed, and 6% (n = 1) reported a neutral response. The mean score for the students was 1.72 (SD = 0.56).

### Effectiveness of Debriefing

Among the AOE respondents, 49% (n = 26) strongly agreed and 47% (n = 25) agreed that the postexercise debriefing effectively identified key learning points, whereas 4% (n = 2) reported neutral responses. The mean score was 1.55 (SD = 0.57). For the student group, 39% (n = 7) strongly agreed, 56% (n = 10) agreed, and 6% (n = 1) reported neutral responses. The mean score was 1.67 (SD = 0.58).

### Impact on Understanding Privacy and Security Principles

Among the AOE respondents, 30% (n = 16) strongly agreed and 45% (n = 24) agreed that the simulation improved their understanding of privacy and cybersecurity principles, whereas 25% (n = 13) reported neutral responses. The mean score was 1.94 (SD = 0.74). Among student participants, 28% (n = 5) strongly agreed, 67% (n = 12) agreed, and 6% (n = 1) reported neutral responses. The mean score was 1.78 (SD = 0.53).

### Understanding Roles and Responsibilities

The simulation enhanced participants’ understanding of roles and responsibilities during a cybersecurity incident. Among the AOE respondents, 43% (n = 23) strongly agreed and 43% (n = 23) agreed, whereas 13% (n = 7) reported neutral responses. The mean score for this group was 1.70 (SD = 0.69).

Among the student participants, 39% (n = 7) strongly agreed and 56% (n = 10) agreed, with 6% (n = 1) reporting neutral responses. The mean score for the student group was 1.67 (SD = 0.58). These findings are further detailed in Tables 2 and 3.

**Table 2.** Summary of Simulation Evaluation Results—AOE Participants

**Statement**	**Mean**	**SD**	**N**
Simulation scenario realism and professional relevance	1.38	0.48	53
Objectives clearly defined	1.53	0.54	53
Postexercise effectively identified key learning points	1.55	0.57	53
Improved understanding of privacy and security principles	1.94	0.74	53
Simulation enhanced my understanding of various roles and responsibilities during a cybersecurity incident	1.70	0.69	53

Abbreviation: AOE, Assembly on Education.

**Table 3.** Summary of Simulation Evaluation Results—HIM Students

**Statement**	**Mean**	**SD**	**N**
Simulation scenario realism and professional relevance	1.94	0.62	18
Objectives were clearly defined	1.72	0.56	18
Debriefing effectively identified key learning points	1.67	0.58	18
Simulation improved my understanding of privacy and security principles	1.78	0.53	18
Simulation enhanced my understanding of various roles and responsibilities during a cybersecurity incident	1.67	0.58	18

Abbreviation: HIM, health information management.

## Discussion

This study evaluated a multistakeholder, simulation-based educational intervention designed to enhance health information and informatics learners’ preparation for cybersecurity incident response. Findings from both professional/educator participants and undergraduate students demonstrated consistently positive perceptions of the simulation across multiple domains, including realism, clarity of objectives, debriefing effectiveness, and understanding of cybersecurity principles and interdisciplinary roles.

### Interpretation of Findings

Participants in both groups reported strong agreement that the simulation scenario was realistic and reflective of plausible health care cybersecurity incidents. These findings are consistent with prior research demonstrating that simulation-based learning enhances perceived realism and engagement by immersing learners in authentic, practice-based scenarios.^20^^,^^22^

Notably, professional and educator participants demonstrated slightly stronger agreement across several survey items compared with students, as reflected by lower mean scores. Differences between groups were modest, but this pattern may reflect greater experience and familiarity with real-world health care environments among professional participants. In contrast, student responses, although still highly positive, exhibited slightly greater variability, which may be associated with differences in prior exposure to cybersecurity concepts and health care operations.

Across both groups, participants also reported that the simulation objectives were clearly defined and that the structured debriefing effectively reinforced key learning points. These findings align with prior studies emphasizing the importance of structured debriefing and guided reflection in simulation-based education to support knowledge integration and critical thinking.^23^ In addition, both groups reported increased understanding of privacy and cybersecurity principles, as well as enhanced awareness of interdisciplinary roles and responsibilities during a cybersecurity incident.

### Contribution to Health Information and Informatics Education

This study contributes to the growing body of literature on experiential learning in health information and informatics education by demonstrating the value of simulation as a practice-based pedagogical approach. Prior studies have shown that interactive simulation methods, including tabletop exercise and scenario-based activities, enhance student engagement and applied learning in health administration and informatics education.^19^^,^^23^

Although existing simulation studies have primarily focused on informatics learners or single-group educational settings, this study extends prior work by incorporating a multistakeholder framework and evaluating the simulation across both professional and academic contexts. This broader design reflects the interdisciplinary nature of real-world cybersecurity response and highlights the importance of integrating administrative, clinical, technical, legal, and vendor perspectives into educational experiences.

The simulation incorporates several pedagogical elements that support experiential learning, including real-time decision-making, interdisciplinary role-based collaboration, integration of vendor perspectives, and structured debriefing. These elements align with key domains in health administration and informatics education, including health information technology, information governance, leadership, and organizational communication. By engaging in scenario-based decision-making, learners are able to bridge the gap between conceptual knowledge and real-world practice.

### Practice-Based Learning and Workforce Preparation

The simulation was intentionally designed to support key competencies required in contemporary health information and informatics practice. These include the ability to analyze cybersecurity threats, apply regulatory frameworks such as HIPAA and HITECH, and demonstrate leadership in coordinating interdisciplinary responses to complex cybersecurity incidents. These competencies align with recent work emphasizing the evolving role of HIM professionals in addressing emerging technologies, data governance, and cybersecurity challenges within the health care workforce.^16^

Simulation-based approaches have been shown to improve confidence, teamwork, and applied decision-making skills among health care learners,^20^ and the findings of this study further support the role of experiential learning in preparing the future workforce. The role-based structure of the simulation enables participants to experience the interconnected nature of administrative, clinical, technical, and legal responsibilities in managing cybersecurity incidents.

This approach reflects the realities of modern health care systems, where effective incident response requires coordination across multiple stakeholders. As a result, simulation serves as a practical tool for preparing learners to function in collaborative, high-stakes environments.

### Practical Implications for Education and Implementation

The findings suggest that simulation-based learning can be effectively implemented across diverse educational settings, including professional development environments and undergraduate classrooms. The adaptability of the simulation allows it to be tailored to different learner levels, class sizes, and instructional formats, including both in-person and virtual delivery. Similar flexibility has been noted in prior tabletop simulation studies, which emphasize scalability and adaptability as key strengths of simulation-based education.^23^

The simulation may also be adapted for online and hybrid learning environments. In virtual settings, participants can be assigned stakeholder roles using videoconferencing breakout rooms, collaborative documents, and shared communication platforms. Scenario updates can be delivered electronically at timed intervals to simulate evolving cybersecurity incidents. Debriefing discussions may be conducted synchronously or asynchronously using discussion boards or reflective assignments. These adaptations allow programs with online or hybrid curricula to provide experiential cybersecurity training while maintaining the collaborative and interdisciplinary nature of the simulation.

However, successful implementation depends on effective facilitation. Instructors play a critical role in guiding discussions, managing group dynamics, and fostering a psychologically safe learning environment that encourages participation and reflection. Providing appropriate presession preparation and scaffolding may be particularly important for learners with limited prior exposure to cybersecurity concepts.

### Limitations

This study has several limitations that should be considered when interpreting the findings. The simulation was conducted within a specific scenario involving a mid-sized acute care hospital, which may limit its generalizability to other health care settings without adaptation. In addition, the evaluation relied on self-reported survey data, reflecting participant perceptions rather than direct measures of learning outcomes. The study did not assess knowledge gain or behavioral change, limiting the ability to evaluate learning outcomes beyond participant perceptions. The effectiveness of the simulation may also vary depending on instructor facilitation, as the quality of the learning experience is influenced by how the simulation is delivered and debriefed. Furthermore, the simulation has not yet been tested across multiple institutions or diverse learner populations, limiting broader applicability. Participant demographic information, such as age, gender, educational attainment, and race/ethnicity, was not collected because the survey was designed primarily to evaluate participant perceptions of the educational activity. Therefore, differences in perceptions across demographic groups could not be examined. Future research should incorporate more rigorous evaluation methods, such as pre- and post-assessments, competency-based rubrics, or mixed-method approaches, and examine implementation across varied educational and organizational contexts to better assess scalability and long-term educational impact.

## Conclusion

This study demonstrates that simulation-based learning offers a valuable and practical approach to preparing health information and informatics learners for the complexities of cybersecurity incident response in health care settings. By immersing participants in realistic, time-sensitive scenarios, the simulation fosters critical thinking, regulatory awareness, interdisciplinary collaboration, and leadership skills essential for modern health care practice.

The findings highlight the potential of simulation to bridge the gap between theoretical instruction and real-world application. As health care systems continue to face increasing cybersecurity threats, incorporating experiential, practice-based learning approaches into educational programs is essential to ensure workforce readiness.

Health information and informatics programs are encouraged to adopt and adapt simulation-based approaches to strengthen student engagement, enhance applied learning, and better prepare future professionals to navigate the evolving challenges of digital health care environments.

## Author Contributions

All authors contributed to the study conception and design. Material preparation, data collection, and analysis were performed by the authors. All authors contributed to manuscript drafting and approved the final version of the manuscript.

## Disclosures

The authors have nothing to disclose.

## Funding

The authors received no funding for this research.

CE Quiz

## Supplementary Material

Supplemental Material

## Appendixes

### Appendix A: Simulation Scenarios and Instructional Materials

The following scenarios were used to guide the tabletop simulation. Each scenario includes a structured description, suggested response actions, and guided discussion questions to support interdisciplinary decision-making and reflective learning.

#### Scenario A1. Ransomware Attack and System-Wide Disruption

##### Scenario Description

A ransomware attack, initiated through a phishing email, spreads across the hospital’s internal network, encrypting data within critical systems. The electronic health record (EHR), laboratory, radiology, and billing platforms become inoperable. Clinical teams are required to transition to paper-based documentation, whereas information technology (IT) and administrative teams work to contain the breach and restore system functionality. Organizational leadership must balance immediate operational response with regulatory compliance, communication strategies, and long-term recovery planning.

##### Suggested Response Actions

Participants are expected to consider and prioritize the following actions:

• Activate the internal cybersecurity incident response team
• Isolate compromised systems to prevent further spread
• Implement downtime procedures for clinical documentation and workflows
• Conduct an initial risk assessment to determine the scope and severity of the breach
• Engage external cybersecurity experts and, if appropriate, law enforcement
• Evaluate breach notification requirements under HIPAA and HITECH
• Develop and initiate a system recovery and business continuity plan

##### Guided Discussion Questions

1. What are the immediate priorities in responding to a ransomware attack in a health care organization?
2. How should communication be coordinated across clinical, administrative, and technical teams?
3. What regulatory obligations must be considered under HIPAA and HITECH?
4. What are the operational and patient safety risks associated with downtime procedures?
5. How can leadership maintain patient safety, organizational transparency, and public trust during the crisis?

#### Scenario A2. Phishing Attack and Patient Portal Compromise

##### Scenario Description

An employee in the billing department inadvertently clicks a malicious link in a phishing email disguised as an internal human resources communication. The attacker gains unauthorized access to the hospital’s network and compromises the patient portal. Patients begin reporting suspicious activity, including altered demographic and clinical information, incorrect laboratory results, and difficulty accessing their accounts. Public concern escalates through social media, prompting the organization to suspend the patient portal and initiate emergency response protocols. Leadership must manage the breach while addressing regulatory, legal, operational, and reputational challenges.

##### Suggested Response Actions

Participants are expected to evaluate and prioritize the following actions:

- Determine the timeline, scope, and extent of unauthorized access
- Suspend or restrict patient portal functionality to prevent further compromise
- Initiate a formal risk assessment and breach investigation
- Implement breach notification procedures in accordance with regulatory requirements
- Communicate transparently with affected patients, staff, and regulatory bodies
- Strengthen cybersecurity controls, including multifactor authentication and antiphishing training

##### Guided Discussion Questions

1. What are the immediate response priorities following a patient portal breach?
2. How should communication with affected patients be structured and delivered?
3. What are the implications of disabling the patient portal for patient engagement and care continuity?
4. What preventive safeguards could have mitigated or prevented this incident?
5. How should leadership balance transparency, legal considerations, and reputational risk?

### Appendix B. Overview of Simulation Implementation and Delivery Procedures

**Table:**

**Component**	**Description**
Presession preparation	Assign stakeholder roles and distribute briefing materials; provide optional prereadings on cybersecurity, HIPAA/HITECH, and crisis leadership
Preparation and orientation	Instructor introduces the scenario and provides role orientation; participants begin team-based analysis and strategy development
Response planning	Teams collaborate to assess the incident, develop response strategies, and make decisions under time constraints
Team presentations	Groups present coordinated response plans and rationale
Facilitated debriefing	Structured reflection on decisions, outcomes, and real-world implications, with emphasis on leadership and interdisciplinary communication
Instructor considerations	Foster a psychologically safe learning environment; encourage reflection; balance guidance with participant autonomy; adapt to class size and delivery format
