# Supplementary material for: Beyond the Firewall: A Multistakeholder Simulation for Cybersecurity Response in Health Information and Informatics Practice
Source: Adv Health Inf Sci Pract. 2026 Aug 12;2(2):eah.000000004. doi: 10.63116/AH.000000004 (PMC13463233; doi:10.63116/AH.000000004)
Supplement: Supplementary file 1 [file Supplementary_Appendix.docx]

**Appendix A. Simulation Scenarios and Instructional Materials**

The following scenarios were used to guide the tabletop simulation. Each scenario includes a structured description, suggested response actions, and guided discussion questions to support interdisciplinary decision-making and reflective learning.

**Scenario A1. Ransomware Attack and System-Wide Disruption**

**Scenario Description**

A ransomware attack, initiated through a phishing email, spreads across the hospital’s internal network, encrypting data within critical systems. The electronic health record (EHR), laboratory, radiology, and billing platforms become inoperable. Clinical teams are required to transition to paper-based documentation, while information technology (IT) and administrative teams work to contain the breach and restore system functionality. Organizational leadership must balance immediate operational response with regulatory compliance, communication strategies, and long-term recovery planning.

**Suggested Response Actions**

Participants are expected to consider and prioritize the following actions:

- Activate the internal cybersecurity incident response team
- Isolate compromised systems to prevent further spread
- Implement downtime procedures for clinical documentation and workflows
- Conduct an initial risk assessment to determine the scope and severity of the breach
- Engage external cybersecurity experts and, if appropriate, law enforcement
- Evaluate breach notification requirements under HIPAA and HITECH
- Develop and initiate a system recovery and business continuity plan

**Guided Discussion Questions**

1. What are the immediate priorities in responding to a ransomware attack in a healthcare organization?
2. How should communication be coordinated across clinical, administrative, and technical teams?
3. What regulatory obligations must be considered under HIPAA and HITECH?
4. What are the operational and patient safety risks associated with downtime procedures?
5. How can leadership maintain patient safety, organizational transparency, and public trust during the crisis?

**Scenario A2. Phishing Attack and Patient Portal Compromise**

**Scenario Description**

An employee in the billing department inadvertently clicks a malicious link in a phishing email disguised as an internal human resources communication. The attacker gains unauthorized access to the hospital’s network and compromises the patient portal. Patients begin reporting suspicious activity, including altered demographic and clinical information, incorrect laboratory results, and difficulty accessing their accounts. Public concern escalates through social media, prompting the organization to suspend the patient portal and initiate emergency response protocols. Leadership must manage the breach while addressing regulatory, legal, operational, and reputational challenges.

**Suggested Response Actions**

Participants are expected to evaluate and prioritize the following actions:

- Determine the timeline, scope, and extent of unauthorized access
- Suspend or restrict patient portal functionality to prevent further compromise
- Initiate a formal risk assessment and breach investigation
- Implement breach notification procedures in accordance with regulatory requirements
- Communicate transparently with affected patients, staff, and regulatory bodies
- Strengthen cybersecurity controls, including multi-factor authentication and anti-phishing training

**Guided Discussion Questions**

1. What are the immediate response priorities following a patient portal breach?
2. How should communication with affected patients be structured and delivered?
3. What are the implications of disabling the patient portal for patient engagement and care continuity?
4. What preventive safeguards could have mitigated or prevented this incident?
5. How should leadership balance transparency, legal considerations, and reputational risk?

**Appendix B. Overview of Simulation Implementation and Delivery Procedures**

| **Component** | **Description** |
| --- | --- |
| **Pre-session Preparation** | Assign stakeholder roles and distribute briefing materials; provide optional pre-readings on cybersecurity, HIPAA/HITECH, and crisis leadership |
| **Preparation and Orientation** | Instructor introduces the scenario and provides role orientation; participants begin team-based analysis and strategy development |
| **Response Planning** | Teams collaborate to assess the incident, develop response strategies, and make decisions under time constraints |
| **Team Presentations** | Groups present coordinated response plans and rationale |
| **Facilitated Debriefing** | Structured reflection on decisions, outcomes, and real-world implications, with emphasis on leadership and interdisciplinary communication |
| **Instructor Considerations** | Foster a psychologically safe learning environment; encourage reflection; balance guidance with participant autonomy; adapt to class size and delivery format |
